# Application of a Surimi-Based Coating to Improve the Quality Attributes of Shrimp during Refrigerated Storage

**DOI:** 10.3390/foods6090076

**Published:** 2017-09-05

**Authors:** Abdulhakim Sharaf Eddin, Reza Tahergorabi

**Affiliations:** Food and Nutritional Sciences program, Department of Family and Consumer Sciences, North Carolina Agricultural and Technical State University, Greensboro, NC 27411, USA; alarrab_sh@yahoo.com

**Keywords:** surimi, shrimp, montmorillonite, quality, coating

## Abstract

Shrimp is a popular seafood throughout the world. However, shrimp is highly perishable due to biochemical, microbiological, or physical changes during postmortem storage. In this study, the effect of a surimi-based coating with and without montmorillonite (MMT) nanoclay on shrimp quality was evaluated during eight days of refrigerator storage. Use of a surimi-based coating resulted in reductions of aerobic plate counts (APC) up to 2 log units. The combined effect of the MMT and coating was observed. Surimi-based coating with MMT resulted in lower APC (*p* < 0.05) toward the end of storage. The application of surimi-based coating with MMT to the shrimp samples improved sensory quality and delayed lipid oxidation and color deterioration during storage time. In general, better texture was observed when coating was applied either with or without MMT. This study suggests that surimi-based coating may improve the quality of shrimp during refrigerated storage.

## 1. Introduction

Shrimp is one of the most desirable crustaceans in the US and the world because it has a unique rich flavor and texture [1]. Shrimp contains many nutrients including protein, minerals, and poly unsaturated fatty acids, that are considered essential for humans’ health, such as docosahexaenoic acids (22:6*n*3, DHA) and eicosapentaenoic acids (20:5*n*3, EPA) [2]. Furthermore, shrimp has a lower level of mercury content compared to other seafood species [1]. However, shrimp is quite a perishable food. The quality of shrimp is influenced by several factors, such as the method of handling, storage condition, and processing time. The shelf life of shrimp is mostly determined by both microbiological and enzymatic spoilage when stored at refrigerator temperature. Shrimp contains large amounts of free amino acids that are contributed to microbiological spoilage [3]. In addition, shrimp may suffer from black spot (melanosis) due to the activity of polyphenol oxidase [4]. It has been reported that 1.2% of the lipids that are located just under the shrimp shell are highly unsaturated phospholipids [5]. Therefore, lipid oxidation and rancid off-flavors may also occur even under refrigeration or freezing conditions [4]. When properly stored, the shelf-life of shrimp at refrigerator temperature may last 1–2 days [2]. As a result, it is necessary to devise a strategy to prevent or slow down quality degradation of shrimp during storage time.

Currently, there is a great interest in the application of natural preservatives in food industry. However, their direct application has some drawbacks, including changes of sensory properties, fast release of active compounds and interacting with other food ingredients. These limitations have directed researchers to adopt coating as a method, alone or in combination with other methods, to increase the shelf-stability of perishable foods such as shrimp. The features of the coating application can contribute to maintain the quality of seafood products, and delay spoilage at low temperature with minimum effects on the characteristic of the product [6].

Coating is defined as a thin layer mainly made from polysaccharides, proteins, and lipids which may protect food from losing its quality and prevent the loss of other important components, in addition to contribution to extend the shelf life. At the same time, coatings are logical vehicles for functional ingredients, such as antibacterial and antioxidant agents [7]. Overall, in an identical manner to polysaccharides, proteins are excellent aroma barriers. Like oil barriers, proteins provide strength and structural integrity, but proteins are not effective moisture barriers. Nanoparticles may be used to overcome this issue. The most well-known nano particles are layered clays, such as montmorillonite (MMT) [8]. MMT has also received great attention from researchers, due to the high bactericidal effects. The layers of MMT could attach to the cellular surface of bacteria, decrease its permeability and kill the bacteria [9]. Additionally, the cation reactions of MMT with the anions in the cell wall will result in bacteria death. Researchers have shown that dispersed MMT decreased the number of *S. aureus* and *E. coli* bacterial colonies [10]. This may provide a synergistic effect for coating to maintain the quality of the product during the storage duration. The main risk of consumer exposure to nanoclays is through migration of nanoparticles or other substances from films or edible coatings into foods which are then eaten. However, migration studies are few in number. The general formula for MMT nanoclay is (Al_1.67_(Mg_0.33_Na_0.33_))Si_4_O_10_(OH)_2xH2O_ with variable quantities of H_2_O and interlayer cations [8]. Aluminum and silicon are normally chosen as markers to follow the MMT migration due to their presence at the highest quantities. Concerning food regulation, to date there is no specific migration limit on aluminum or silicon. However, the European Food Safety Authority has issued an opinion on the safety of aluminum from dietary intake and has established a tolerable weekly intake of 1 mg/kg of body weight/week (i.e., 60 mg/week/adult). This is, in the worst case approach, equivalent to 8.6 mg of aluminum/kg of food, which is much higher than the values obtained in the edible coating [11]. A fish protein isolate coating with MMT on fresh-cut pear was tested and the application of the coating resulted in lower microbial populations, weight loss, and improvement of texture, color, and pH [12]. Similarly, it has been shown that Whitemouth croaker protein isolate and organo-clay-based edible coatings could improve the quality attributes of fresh-cut Formosa papaya compared to uncoated samples [13].

Surimi is a washed fish muscle protein that is mixed with cryoprotectants [14]. The gelling property of surimi makes it a good candidate for seafood-based products. Few researchers have fabricated protein films from surimi proteins [15,16,17]. However, there has been no attempt to study the effect of coating made from surimi on the quality of food products. A better understanding of the quality changes of shrimp coated with surimi could be helpful for elucidating the role of this coating in shelf-life extension and development of new surimi-based products. Therefore, the objectives of this study were to evaluate the effect of a surimi-based coating from Alaska Pollock with or without MMT nanoclay on (1) physico-chemical (pH, lipid oxidation, texture, color) properties; (2) microbial quality; and (3) sensory and melanosis characteristics of shrimp during the storage time in a refrigerator.

## 2. Materials and Methods

### 2.1. Materials

Natural montmorillonite (MMT) nanoclay was purchased from Nanomer (St. Louis, MO, USA). According to the manufacturer, the MMT product had a moisture content below 2.0%, a particle size of 10% smaller than 2.00 μm, 50% smaller than 6.00 μm, and 90% smaller than 13.0 μm. Glycerol was acquired from Fisher Scientific (Fair Lawn, NJ, USA). Trident Seafoods Corp. (Seattle, WA, USA) provided the frozen blocks of Alaskan Pollock grade A surimi. Medium-sized uncooked frozen shrimp were procured from a local market. Frozen shrimp had been treated with phosphate according to the information provided on the label of product. Phosphate is added to shrimp before freezing as a cryoprotectant. It also helps to increase water binding by increasing the pH [18]. Shrimp were kept in an ice container until arrival at the laboratory and stored at −18 ± 1 °C until used. Shrimp samples were coated by a surimi protein coat, and tested for physicochemical, microbiological, and sensory evaluations during eight days of storage at 4 ± 1 °C.

### 2.2. Preparation of Surimi Protein Solution

Surimi coating solution was prepared according to the method previously reported in the literature with slight modification [13]. Distilled, deionized water was mixed with surimi protein and the final protein concentrations was adjusted to 2% (*w/v*). The mixture was homogenized for 5 min using a general laboratory homogenizer (Omni GLH, Oxford, CT, USA), then dispersed at 30 °C in a thermostatic ultrasonic bath (Bransonic, CPX3800H, Danbury, CT, USA) for 20 min to hydrate the protein. NaOH (1N) was used to raise the pH to 11.2. The temperature of the solution was adjusted to 80 °C, and 10.5 g of dissolved glycerol in distilled water was added to the solution, then the solution was homogenized for 5 min. For the preparation of surimi protein solution with MMT, 3% of MMT was added after elevating the solution temperature to 80 °C. These coating solutions were used for coating the shrimp.

### 2.3. Shrimp Coating

Shrimp samples were placed in styrofoam trays and divided into three treatments: Treatment 1 (T1, uncoated-control), treatment 2 (T2, coated with surimi protein), and treatment 3 (T3, coated with surimi protein and MMT). Samples in T2, and T3 were immersed in the coating solution for 5 min, and then they were dried by placing them on sieves for 2–3 min at room temperature. The samples in all treatments were stored in a refrigerator at 4 ± 1 °C for eight days.

### 2.4. Instrumental Color

A Minolta Chroma Meter CR-400/410 colorimeter (Minolta Camera Co. Ltd., Osaka, Japan) was used to determine the color properties of shrimp. The L*a*b* tristimulus color values were determined using the CIE (Commission Internationale d’Eclairage of France) method. Three readings for three different places on the surface of shrimp for each sample were measured, and then the mean for these readings was calculated [14].

### 2.5. pH Value

The pH value was measured by using a pH meter (Accumet, AB 150, Singapore). The probe was calibrated before each measurement. One hundred milliliters of deionized water were added to 20 g of shrimp sample and homogenized for 1 min. The pH was determined after 5 min [19].

### 2.6. Texture Properties

Texture profile analysis (TPA) was applied to determine the shrimp samples textural properties using a texture analyzer (Model TAXT2, Texture Technologies Corp, Scarsdale, NY, USA). Different textural parameters are evaluated using the empirical method of TPA, including hardness, springiness, cohesiveness, gumminess, chewiness, and resilience [20].

### 2.7. TBARS Value

Lipid oxidation of shrimp samples were determined by using TBARS assay of malondialdehyde (MDA) according to our previously-reported method [21].

### 2.8. Aerobic Plate Counts

All samples were subjected to microbiological analysis. One gram of shrimp muscles were added into peptone water (0.1%), then the samples were homogenized for 1 min, and the samples were then serially diluted in 9 mL peptone water (0.1%). The aerobic plate counts were enumerated by spread-plating of 1 mL of sample solution on sterile Petri-plates containing plate count agar (Difco Laboratories, Detroit, MI, USA). Plates were incubated for 24 h at 35 ± 1 °C [22].

### 2.9. Melanosis

A 10-point scoring test was used to evaluate black spots on the surface of shrimp. The melanosis was rated from 0 to 10. If there is no black spot on the surface of shrimp, then it is rated as zero (0), while 10 indicates that 80–100% of the shrimp’s surface is covered by black spots [19].

### 2.10. Sensory Analysis

Over 100 untrained panelists volunteered to evaluate the shrimps stored in a refrigerator on days one and eight. Each panelist was designated to one sensory station. Raw samples which were labeled by random three digit codes were placed on trays and served to the panelists. Panelists were asked to score appearance, odor, and texture using a nine-point scale, where 9 was the highest quality score, 1 was the lowest, and 5 was an acceptable level [23].

### 2.11. Statistical Analysis

Each experiment was performed three times, independently (*n* = 3). For each experiment, the mean and standard deviation of the result was recorded; then an analysis of variance using two-way ANOVA was conducted (SAS version 16.0, SAS Institute, Cary, NC, USA). Differences in the mean values of the results of experiments were calculated using Tukey’s test, and the criterion for labeling a difference as significant was *p* < 0.05.

## 3. Results

### 3.1. Instrumental Color

The tristimulus color values of shrimp samples are shown in Table 1. In biodegradable films and coatings, color is an important property because it could influence consumer purchasing decisions [24]. The application of surimi-based coating with or without MMT resulted in significanly higher (*p* < 0.05) L*, a*, and b* values than the control samples. On the other hand, storage time had no significant effect (*p* > 0.05) on L*, a* and b* values of shrimp samples of different treatments. Researchers [25] noted that three months of frozen storage of pink salmon coated with protein-based coating had no significant effect on the color properties of the fillets.

### 3.2. pH Value

**Figure 1:**
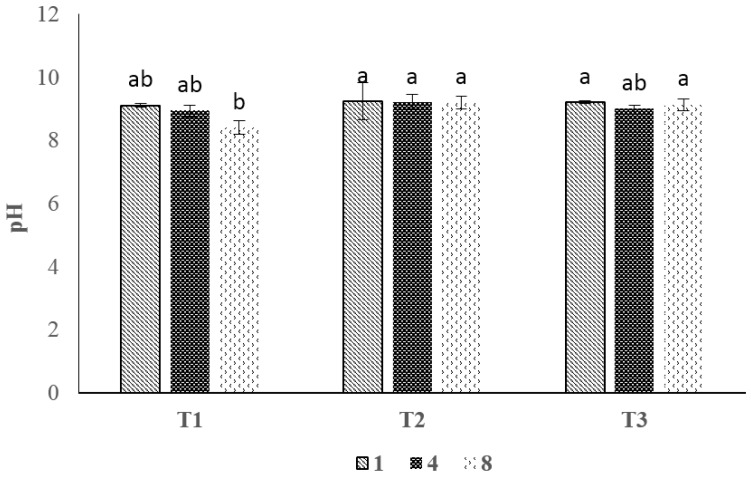
The effect of surimi-based coating on pH of shrimp during storage at 4 ± 1 °C. Data are given as mean values ± standard deviation (*n* = 3). Different letters on the top of data bars indicate significant differences (Tukey’s test, *p* < 0.05) between mean values. T1, control; T2 surimi-based coating without MMT; T3, surimi-based coating with MMT.

Figure 1 shows the pH values of the uncoated controls (T1) and the surimi-based coatings without (T2) and with MMT (T3). The initial pH of shrimp for uncoated samples was recorded as 9.1. Similarly, the pH values of 9.10–9.27 at first analysis was reported in another study which were high for shrimp species [18], compared to the normal pH values of shrimp (i.e., 6.5–7.0) [26,27], and this may be due to the use of phosphate before the freezing process, as mentioned in Section 2.1. However, in their study, during storage, pH of the samples decreased steadily with time for all studied groups. While in our study, the pH values for T1 and T2 were either similar or slightly higher than those of control samples.

### 3.3. Texture Properties

**Figure 2:**
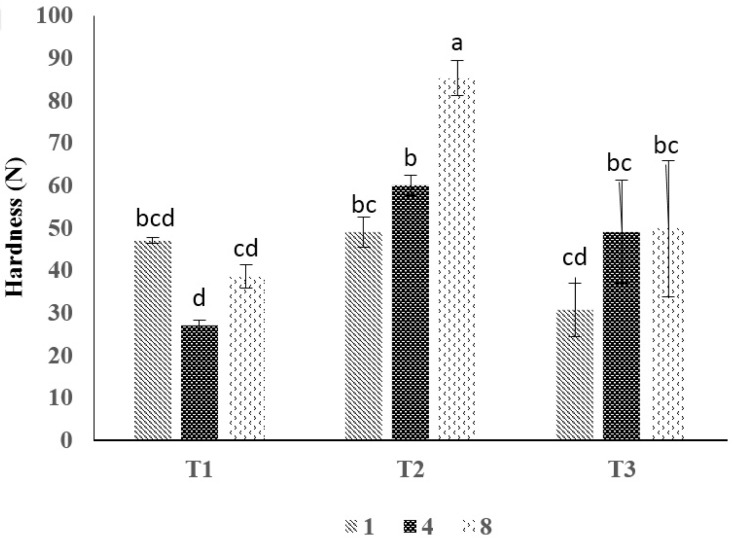
The effect of surimi-based coating on hardness of shrimp during storage at 4 ± 1 °C. Data are given as mean values ± standard deviation (*n* = 3). Different letters on the top of data bars indicate significant differences (Tukey’s test, *p* < 0.05) between mean values. T1, control; T2 surimi-based coating without MMT; T3, surimi-based coating with MMT.

Six factors are determined using texture profile analysis (TPA) including hardness, springiness, cohesiveness, gumminess, chewiness, and resilience. Table 2 and Figure 2 show the effect of surimi-based coating on the texture of shrimp. There was no significant difference for resilience, cohesiveness, and springiness of the control and surimi-based coated shrimp samples with or without MMT during the storage time (*p* > 0.05). Surimi-based coated shrimp possessed higher gumminess and chewiness (*p* < 0.05). Texture of shrimp samples were not deteriorated during storage time. However, textural scores for control samples were numerically slightly lower than those of treated samples.

### 3.4. TBARS Value

**Figure 3:**
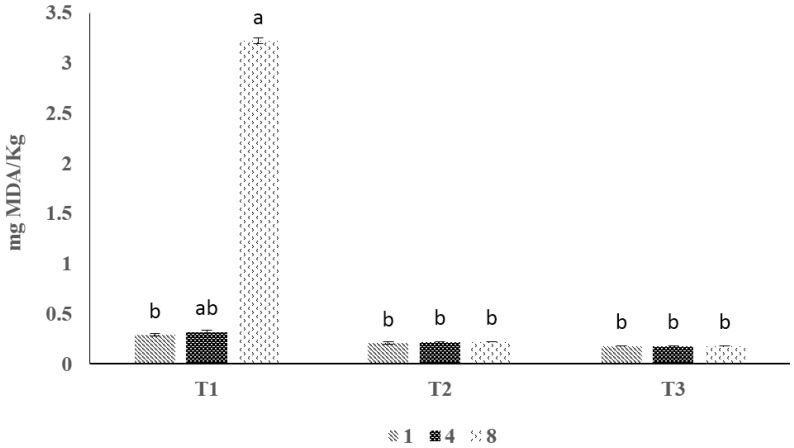
The effect of surimi-based coating on TBARS of shrimp during storage at 4 ± 1 °C. Data are given as mean values ± standard deviation (*n* = 3). Different letters on the top of data bars indicate significant differences (Tukey’s test, *p* < 0.05) between mean values. T1, control; T2 surimi-based coating without MMT; T3, surimi-based coating with MMT.

TBARS values of the shrimp without and with surimi-based coating are illustrated in Figure 3. Initial TBARS value of shrimp meat without coating was 0.32 mg MDA/kg. It increased continuously throughout the refrigerated storage (*p* < 0.05). This increment was only noticed for control (un-coated) samples. This result was similar to the results from other researchers [28,29,30].

### 3.5. Aerobic Plate Counts

**Figure 4:**
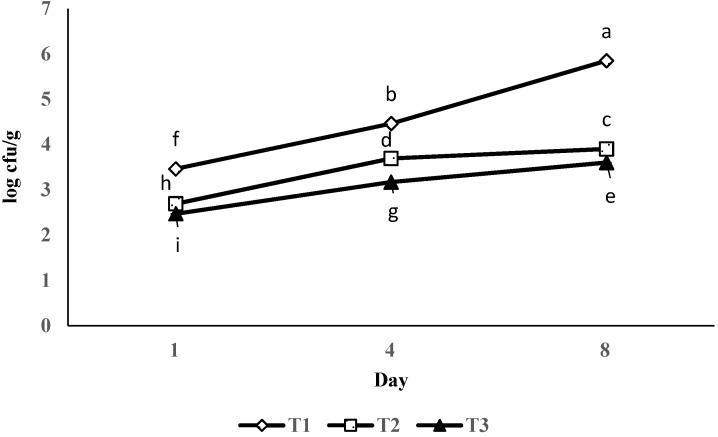
The effect of surimi-based coating on aerobic plate count of shrimp during storage at 4 ± 1 °C. Data are given as mean values ± standard deviation (*n* = 3). Different letters on the top of data bars indicate significant differences (Tukey’s test, *p* < 0.05) between mean values. T1, control; T2 surimi-based coating without MMT; T3, surimi-based coating with MMT.

Counts of bacterial population in coated and uncoated control samples are shown in Figure 4. APC of uncoated shrimp meat was 3.46 log cfu/g, which increased sifnificantly (*p* < 0.05) over time. Shrimp meat in the control group showed higher APC than the surimi-based coated samples. The APC levels of the coated and uncoated samples did not reach less than 7 log cfu/g, which is the limit for the onset of spoilage established for fresh shrimp by the International Commision on Microbiological Specifications for Foods [31] even after eight days of storage.

### 3.6. Melanosis

The melanosis score of coated and uncoated shrimp samples are shown in Table 3. At day 1, all samples had no melanosis (score = 0). At the end of storage time, up to 20% of the shrimp’s surface in the control group (T1) was covered by black spots. In another study, Nirmal and Benjakul [19] noticed severe melanosis after four days of storage in control shrimp samples, and even those samples which were coated with sodium metabisulfite. However, in our study, no melanosis was noticable in surimi-based coated shrimp samples with or without MMT.

### 3.7. Sensory Analysis

Table 4 shows the sensory scores of coated and uncoated shrimp. In general, surimi-based coated shrimps with (T3) and without MMT (T2) showed significantly higher (*p* < 0.05) scores for sesnsory evaluation compared to the control samples (T1).The lowest sensory scores for appearance, odor, and texture were found in control samples at the end of the storage period due to a slight formation of black spot, whereas no melanosis was observed on surimi-based coated shrimp with or without MMT during the storage period. The appearance scores in T2 and T3 were close to each other. The results of sensory and melanosis were supportive of each other.

## 4. Discussion

### 4.1. Instrumental Color

Previously, few researchers prepared protein-based films from dark and white muscle fish and reported that when films were prepared from white muscle showed the highest L* values (*p* < 0.05) [32]. This is in agreement with our results since the surimi-based coating in this study prepared from washed Alaska Pollock white muscle. The increase in b* values might be related to the alkaline conditon that the surimi-based coating was prepared in this study. Similary, surimi-based films from bigeye snapper at various pH levels was prepared [16]. In their study, the films that were prepared at alkaline pH had higher L* and b* values. Increased b* (yellowness) values may also be contributed to MMT incorporation in the surmi-based coating. Higher yellowness in the bio-nanocomposite films prepared with different concentrations of MMT was reported in previous studies [33].

### 4.2. pH Value

Shrimp meat coated with surimi-based coating with or without MMT had similar or slightly higher pH values, as compared with the control group (*p* < 0.05). This might be contributed to the high pH of the surimi-based coating solution, resulting from NaOH incorporation into formulation. During the storage time the pH values were constantly high (*p* > 0.05). In contrast, researchers [34] found lower pH when applied chitosan coating on shrimp. This lower pH was due to acetic acid incorporation into the coating formulation. Normally, during the storage time, the pH is increased due to alkaline compounds that are produced from bacterial activity [19]. However, this trend was not detected in our study. This is in agreement with the aerobic plate counts of the coated shrimp samples during the storage time.

### 4.3. Texture Properties

A higher hardness was found in surimi-based coated shrimp (T2), compared with those of controls (T1) and surimi-based coating with MMT (T3). Generally, the lower values for control samples might be related to proteolytic activity of endogenous or microbial proteinases and collagenase. The lower bacterial population is correlated with higher hardness values of coated shrimp. This is due to proteinases that are produced by spoilage microorganisms which hydrolyze the muscle protein. Furthermore, it has been suggested that the differences in hardness might be also related to different protein structure of shrimp [35]. This could be translated to the quality of shrimp, as well as the consumer acceptance. Myofibrillar protein might be also affected by ice during the frozen storage. However, deep water pink shrimp have shown differences in hardness and elasticity during frozen storage [36]. These results are in agreement with the present study.

### 4.4. TBARS Value

The rise in the TBARS value of the control samples might contribute to the unsaturated fatty acids oxidation and the partial dehydration of shrimp. TBARS values for all treatments in the present study did not reach the limit value for perceived undesirable rancid flavor and odor reported in the literature for fish. Up to 5 mg MDA/kg has been reported as the maximum level of TBARS which indicates the good quality of aquatic food products. However, even up to 8 mg MDA/kg is considered safe to eat for seafood products. In the present study, TBARS for all of the samples were much lower than the proposed limit. However, the lower TBARS values were noticeable when surimi-based coating with or without MMT were used. This might be due to decreased transparency of coated shrimp samples. Light transmission of fish protein films and coatings is low and this suggests that films could prevent the lipid oxidation induced in a food system. The result was in agreement with other researchers [14], who reported films that are made of protein are excellent UV barriers due to their large amount of aromatic amino acids which absorb UV light. In this study incorporation of MMT had no significant effect (*p* > 0.05). Therefore, surimi-based coating could be a viable solution to prevent lipid oxidation over the time of storage.

### 4.5. Aerobic Plate Counts

At the end of refrigerated storage, all treatments, including the control group, did not surpass 5.85 log cfu/g. This is in agreement with other scientists [37] who reported the maximum count of 5.5 log cfu/g for all the shrimp samples coated or uncoated with chitosan. It suggests the benefit of low-temperature storage of shrimp. On the other hand, it was shown that if fish is gutted, the shelf life will be extended since fewer bacterial populations invade the fish during the refrigerated storage. Shrimp belong to crustacean speccies and have a slim gut. All these factors may influnece the presence and growth of bacteira in shrimp muscle [38]. MMT incorporation into surimi-based coating exhibited antibacterial effects as measured by APC up to eight days. It was reported that MMT nanoclay led to a decrease in the number of *S. aureus* and *E. coli* bacterial colonies which evidences the antibacterial effect of MMT [10]. The layers of MMT could attach to the cellular surface of bacteria, decrease its permeability, and kill the bacteria. This is due to the attachment of MMT layers to the cell membrane of the microbe; the cation reactions of MMT with the anions in the cell wall will result in bacteria death. As a result, reductions of 1–2.25 log cfu/g in APC were observed with the surimi-based coating application as compared with the control throughout the storage.

### 4.6. Melanosis

Shrimp suffer from discoloration (black spot) that is caused by the activity of polyphenol oxidase that oxidize phenols to quinines, these spots start to form on the surface of shrimp after harvesting within a few hours, if the shrimp was kept without refrigeration. Polyphenol oxidase enzyme can catalyze the first step in melanosis processing and can stay active after harvest even though shrimp are frozen or cooked. Storage at low temperature can slow down this reaction, but cannot prevent it [39]. Formation of melanosis is one of the factors that influence the market value of shrimp and crustaceans. On the basis of the results of melanosis, surimi-based coating with or without MMT could prevent melanosis formation.

### 4.7. Sensory Analysis

The appearance scores in T2 and T3 were close to each other. The results of sensory and melanosis were supportive of each other.

## 5. Conclusions

In this study, a surimi-based coating and MMT nanoclay could reduce microbial growth, melanosis, TBARS values and loss of firmness of shrimp samples during eight days of storage compared to uncoated samples. This suggests that surimi protein coating with MMT could be a viable solution to maintain the quality and reduce the losses of shrimp meat.

## Figures and Tables

**Table 1:** The effect of surimi-based coating on the values of L*, a* and b* of shrimp during storage at 4 ± 1 °C.

	Treatments		
Days	T1	T2	T3
L*			
1	41.71 ± 0.65 ^b,c,d^	44.63 ± 0.59 ^a,b,c^	48.91 ± 2.16 ^a^
4	40.92 ± 2.29 ^c,d^	46.11 ± 2.13 ^a^	44.98 ± 2.48 ^a,b,c^
8	39.78 ± 1.00 ^c^	47.89 ± 1.02 ^a^	46.08 ± 0.39 ^a,b^
a*			
1	−1.21 ± 0.72 ^c^	1.16 ± 0.36 ^a,b,c^	0.83 ± 0.13 ^b,c^
4	0.79 ± 0.33 ^b,c^	3.08 ± 2.36 ^a,b^	2.29 ± 1.92 ^a,b,c^
8	1.62 ± 0.97 ^a,b,c^	4.93 ± 2.10 ^a^	1.75 ± 0.65 ^a,b,c^
b*			
1	−0.93 ± 2.50 ^d^	5.46 ± 0.82 ^a^	1.37 ± 0.45 ^b,c,d^
4	1.06 ± 0.88 ^c,d^	2.7 ± 1.27 ^a,b,c,d^	3.74 ± 2.38 ^a,b,c^
8	6.26 ± 1.49 ^a^	4.32 ± 0.15 ^a,b,c^	5.22 ± 0.93 ^a,b^

Data are given as mean values ± standard deviation (*n* = 3). Different letters within the same row indicate significant differences (Tukey’s test, *p* < 0.05) between mean values. T1, control; T2, surimi-based coating without MMT; T3, surimi-based coating with MMT.

**Table 2:** The effect of surimi-based coating on texture profile analysis of shrimp during storage at 4 ± 1 °C.

	Treatments		
Day	T1	T2	T3
Resilience			
1	0.3 ± 0.01 ^a^	0.22 ± 0.02 ^a,b^	0.16 ± 0.02 ^b^
4	0.21 ± 0.04 ^a,b^	0.25 ± 0.02 ^a,b^	0.19 ± 0.03 ^a,b^
8	0.2 ± 0.04 ^a,b^	0.27 ± 0.08 ^a,b^	0.19 ± 0.04 ^a,b^
Cohesiveness			
1	0.38 ± 0.07 ^a,b^	0.38 ± 0.03 ^a,b^	0.3 ± 0.02 ^b^
4	0.34 ± 0.01 ^a,b^	0.43 ± 0.03 ^a^	0.36 ± 0.05 ^a,b^
8	0.34 ± 0.05 ^a,b^	0.38 ± 0.02 ^a,b^	0.35 ± 0.07 ^a,b^
Springiness			
1	0.51 ± 0.05 ^a^	0.61 ± 0.12 ^a^	0.48 ± 0.04 ^a^
4	0.55 ± 0.09 ^a^	0.58 ± 0.09 ^a^	0.59 ± 0.02 ^a^
8	0.47 ± 0.01 ^a^	0.64 ± 0.03 ^a^	0.57 ± 0.05 ^a^
Gumminess			
1	16.44 ± 5.54 ^b,c^	18.78 ± 2.84 ^b,c^	9.15 ± 1.37 ^c^
4	17.84 ± 1.20 ^b,c^	26.04 ± 2.30 ^a,b^	18.05 ± 6.56 ^b,c^
8	14.5 ± 0.50 ^b,c^	36.01 ± 0.56 ^a^	18.07 ± 8.89 ^b,c^
Chewiness			
1	6.47 ± 0.85 ^b^	11.48 ± 2.78 ^a,b^	4.94 ± 1.41 ^b^
4	8.02 ± 3.16 ^b^	14.93 ± 1.80 ^a,b^	10.7 ± 4.14 ^a,b^
8	6.63 ± 0.21 ^b^	25.42 ± 16.17 ^a^	13.92 ± 4.25 ^a,b^

Data are given as mean values ± standard deviation (*n* = 3). Different letters within the same row indicate significant differences (Tukey’s Test, *p* < 0.05) between mean values. T1, control; T2 surimi-based coating without MMT; T3, surimi-based coating with MMT.

**Table 3:** The effect of surimi-based coating on melanosis of shrimp during storage at 4 ± 1 °C.

	Treatments		
Days	T1	T2	T3
1	0.0 ± 0.0 ^b^	0.0 ± 0.0 ^b^	0.0 ± 0.0 ^b^
8	2.0 ± 0.0 ^a^	0.0 ± 0.0 ^b^	0.0 ± 0.0 ^b^

Data are given as mean values ± standard deviation (*n* = 3). Different letters within the same row indicate significant differences (Tukey’s Test, *p* < 0.05) between mean values. T1, control; T2 surimi-based coating without MMT; T3, surimi-based coating with MMT.

**Table 4:** The effect of surimi-based coating on sensory scores of shrimp during storage at 4 ± 1 °C.

	Treatments		
Days	T1	T2	T3
	Appearance		
1	7.0 ± 0 ^a^	7.0 ± 0 ^a^	7.4 ± 0.68 ^a^
8	4.67 ± 0.12 ^b^	6.73 ± 0.25 ^a^	7.0 ± 0 ^a^
	Odor		
1	7.0 ± 0 ^a,b^	7.0 ± 0 ^a,b^	7.3 ± 0.26 ^a^
8	5.83 ± 0.29 ^c^	6.5 ± 0.5 ^bc^	7.0 ± 0 ^a,b^
	Texture		
1	7.0 ± 0 ^a,b^	7.0 ± 0 ^a,b^	7.12 ± 0.11 ^a^
8	5.67 ± 0.29 ^c^	6.73 ± 0.25 ^b^	7.0 ± 0 ^a,b^

Data are given as mean values ± standard deviation (*n* = 3). Different letters within the same row indicate significant differences (Tukey’s test, *p* < 0.05) between mean values. T1, control; T2 surimi-based coating without MMT; T3, surimi-based coating with MMT.
